# Innovative CMOS-fabricated dielectrophoretic chip: application of 3D TiN nano-electrode arrays with adjustable electrode spacing in sperm capture

**DOI:** 10.3389/fbioe.2025.1565743

**Published:** 2025-03-27

**Authors:** I-Hsuan Liao, Jeng-Huei Shiau, Kuan-Ru Chou, Chun-Lung Lien, Chao-Min Cheng

**Affiliations:** ^1^ Institute of Biomedical Engineering, National Tsing Hua University, Hsinchu, Taiwan; ^2^ NEAT Biotech Inc., Hsinchu, Taiwan

**Keywords:** dielectrophoresis, nano-electrode arrays, CMOS-fabricated, adjustable electrode spacing, boar sperm

## Abstract

**Introduction:** Dielectrophoresis has been considered an effective method for particle sorting, and scientists have used it to manipulate sperms. However, due to the limitations of the chip fabrication, the conventional dielectrophoresis chip would face several issues such as low throughput, Joule heating, and insufficient dielectrophoretic force in terms of practical applications. To overcome these limitations, we have developed 3D TiN nano-electrode arrays through employing CMOS technology. The electrodes were scaled down to the nanometer scale, and an insulating layer was used to isolate large areas of wiring, thereby reducing the impact of Joule heating on sperms while simultaneously enhancing the electric field strength. Compared to conventional micro-electrodes, the exposed area of micro-electrodes per unit area has been approximately 840 times larger than that of our nano-electrodes, while the electric field strength of nanoelectrodes has been about 5 times higher than that of micro-electrodes.

**Methods:** Our experimental results showed that variations in either applied voltages or applied frequencies with the capacity of adjustable electrode spacing significantly influenced sperm capture efficiency.

**Results and discussion:** Notably, the sperm capture “net” with different sizes could be created by adjusting electrode spacing, providing a variety of sperm capture net configurations that could be used to examine sperm capture efficiency. The optimal sperm capture condition would be a capture space of 70 µm, an applied voltage of 20 Vpp, and an applied frequency of 3 MHz. Under this condition, the sperm capture efficiency could reach 59.98% ± 0.93%, demonstrating a promising capture efficiency for motile sperm. In addition, the throughput of single dielectrophoresis chip could reach 6 mL/h, and multiple chips could be operated simultaneously according to the demand.

## Introduction

In recent years, many studies have pointed out that dielectrophoretic forces have the ability to manipulate particles for sorting purposes, such as in the separation of cells or bacteria (Tran, 2015; Páez-Avilés et al., 2016; Farasat et al., 2022). The advantages of dielectrophoretic separation technology, including rapid processing, label-free operation, and simplicity, have led many scientists to apply it to sperm capture and selection processes as a means of selecting the high-quality sperms, aiding in sex selection, and improving fertilization rates (Huang et al., 2020; Dararatana et al., 2015; Eijkeren 2011; Wongtawan et al., 2020). However, due to sperm processing time constraints, throughput is a frequent challenge that limits practical application. Various dielectrophoresis-based microfluidic devices have been developed using different materials and fabrication methods (Zhang et al., 2019). Conventional dielectrophoresis chips typically use electrodes at the micrometer scale, such as those based on MEMS processes. To enhance their capture capability, researchers have designed various electrode patterns to increase the effective area. However, these designs often require sperms to flow through channel-like structures for capture (Zhang et al., 2019), which can limit device throughput. Additionally, generating sufficient dielectrophoretic force to capture sperms usually requires applying high voltages, which may cause Joule heating when large-area electrodes come into contact with the medium. This interference weakens the dielectrophoretic force and can even lead to sperm death. Studies have shown that when the temperature rises to 40°C, sperm motility parameters drop to zero (Li et al., 2023), and excessive heat can result in sperm death. Although the development of insulator-based dielectrophoresis (iDEP) has partially mitigated the negative effects of Joule heating, the presence of insulating layers necessitates extremely high voltages to capture biological entities (Gallo-Villanueva et al., 2014). Furthermore, previous literatures appear to provide limited direct data regarding sperm capture efficiency. Our study has demonstrated that the newly developed 3D TiN nano-electrode arrays has overcome the throughput limitations of conventional channel-based electrode designs while significantly reducing the adverse effects of Joule heating. Additionally, its excellent sperm capture capability would offer possibilities for further sperm selection and processing.

The 3D TiN nano-electrode arrays used in this study are based on CMOS process technology, reducing electrode size to the nanoscale and utilizing a high-resistance electrode material. This effectively minimizes the exposed electrode area while maintaining biocompatibility, significantly reducing the generation of Joule heat. Additionally, an insulating layer is used to isolate the wires, further minimizing the negative impact of Joule heat on sperm capture and reducing potential damage to the sperms. Supplementary Figure S1 provides a schematic comparison of the effects of Joule heat in micro-electrode and nano-electrode devices. In terms of electric field intensity, the nano-electrode enhances the field strength by about five times that of micro-electrode (Lien and Yuan, 2019). Because micro-electrodes only generate dielectrophoretic forces at the corners of large-area electrodes, the unit effective electric field area in our nano-electrode arrays chip is much larger (Lien and Yuan, 2019). With these improvements, the 3D TiN nano-electrode arrays achieve excellent capture results at lower applied voltages. Regarding the electrode arrays design, the chip we previously used employed a fixed electrode spacing design, with the nano-electrode arrays capturing sperms within an area of about 15 μm (Lu et al., 2024). However, such a fixed structure may limit the further improvement of sperm capture efficiency. In contrast, the current chip we used features adjustable electrode spacing, allowing for optimization based on the size requirements of sperms from different species, further enhancing capture efficiency.

The principle of dielectrophoretic particle separation is based on the difference in the polarization ability between the particles and the medium, achieved by manipulating the particles to move in a specific direction for separation. According to the dielectrophoretic formulas (Formulas 1–3), the main factors affecting the magnitude of the dielectrophoretic force include: the relative dielectric constants of the particle and medium ( $\varepsilon_{p}$ , $\varepsilon_{m}$ ), the dielectric constants ( $\varepsilon_{p}^{*}$ , $\varepsilon_{m}^{*}$ ), conductivity ( $\sigma_{p}$ , $\sigma_{m}$ ), particle radius (r), the root mean square of the electric field gradient ( ${\left(\nabla E\right)}_{RMS}^{2}$ ), and the Clausius–Mossotti Factor (CM factor). In Formula 1, $\varepsilon_{0}$ is the vacuum permittivity. The dielectric constant is related to the particle’s polarization ability, and the CM factor determines the direction of particle movement. Formula 2 further describes the relationship between the polarization degree of the particles and the surrounding medium and can be used to predict particle behavior in a dielectrophoretic field. When a particle’s polarization ability is greater than that of the medium (i.e., $\varepsilon_{p}^{*}>\varepsilon_{m}^{*}$ )), the particles will be attracted to and move toward regions with denser electric field lines; conversely, when the particle’s polarization ability is lower than that of the medium (i.e., $\varepsilon_{p}^{*}<\varepsilon_{m}^{*}$ ), the particles will be repelled and move away from regions with denser electric field lines (Pethig, 2010). Additionally, the formula also shows that frequency affects the difference in the polarization ability between the particles and the medium, further influencing the particles’ motion behavior.

$$F_{\text{DEP}}=2\pi \varepsilon_{m}\varepsilon_{0}r_{\text{ext}}^{3}\text{Re}\left[\text{CM}\left(f\right)\right]{\left(\nabla E\right)}_{\text{RMS}}^{2}$$  (1)

$$\text{CM}\left(f\right)=\left[\frac{\varepsilon_{p}^{*}-\varepsilon_{m}^{*}}{\varepsilon_{p}^{*}+{2\varepsilon }_{m}^{*}}\right]$$  (2)

$$\varepsilon_{p}^{*}=\varepsilon_{p}\varepsilon_{0}-j\frac{\sigma_{p}}{\pi f},\varepsilon_{m}^{*}=\varepsilon_{m}\varepsilon_{0}-j\frac{\sigma_{m}}{\pi f}$$ (3)

When applying the theory to the context of dielectrophoretic sperm capture, the capture efficiency is influenced by various factors such as the dielectric constants of the sperms and medium, the size of the sperms, and the applied frequencies and voltages. In the case of the dead sperms, the integrity of the cell membrane is compromised, causing the dielectric constant of the sperms to align closely with that of the medium. This results in the molecular term in Formula 2 approaching zero, making the CM factor approach zero as well. As a result, the dielectrophoretic force acting on the dead sperms is extremely weak, and it cannot exert a significant effect, ultimately rendering the dead sperms incapable of being effectively captured. In contrast, the live sperms, with an intact cell membrane, can benefit from the selection of an appropriate medium that creates a significant difference in dielectric constants between the sperms and the medium. This maximizes the CM factor while minimizing damage to the sperms, allowing the live sperms to be effectively influenced by the dielectrophoretic force and captured efficiently.

**FIGURE 1:**
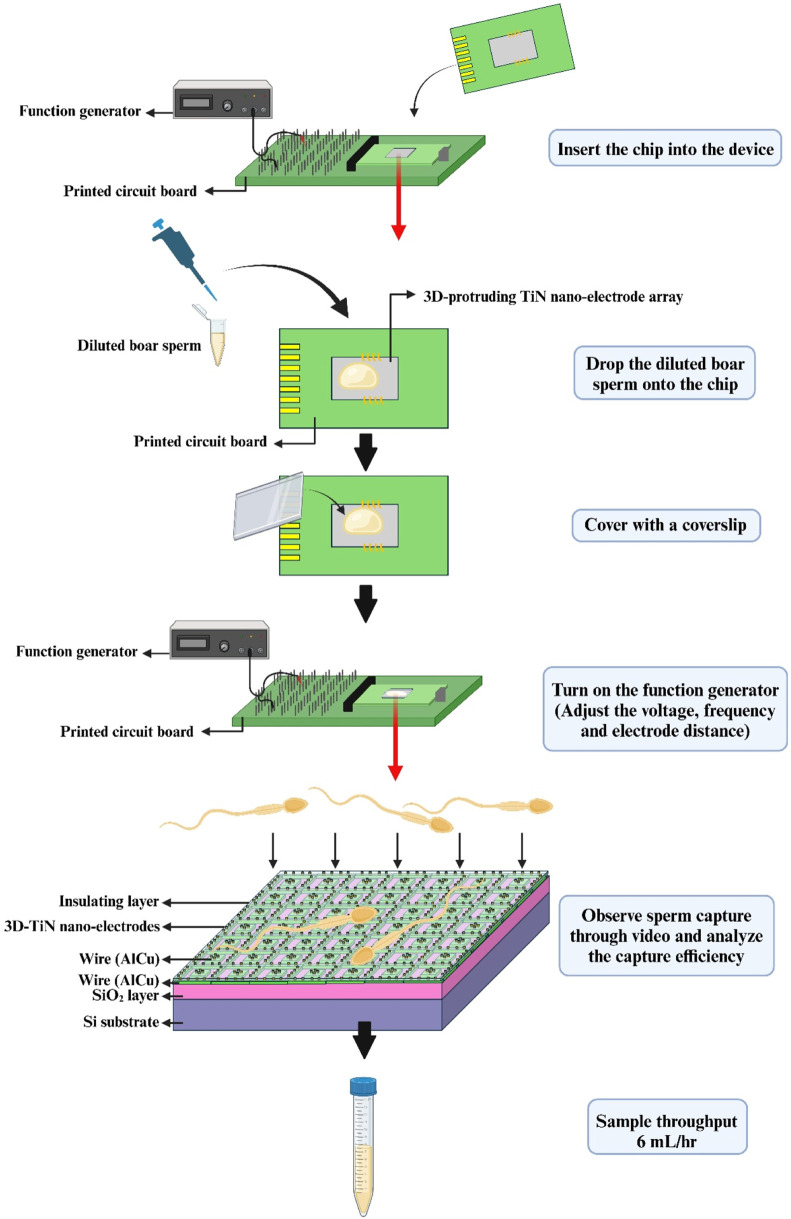
Schematic diagram of the experimental process.

In this study, we focused on observing the sperm capture efficiency under different applied voltages, applied frequencies, and electrode spacing conditions. The experimental process is shown in Figure 1. First, as the applied voltage increased, sperm capture efficiency also increased, suggesting that a stronger electric field positively impacted sperm capture. Second, higher applied frequencies had a positive effect on sperm capture efficiency, while sperm capture efficiency was relatively lower under low-frequency conditions. Additionally, adjusting the electrode spacing produced the “net” with different sizes for capturing sperms. Proper electrode spacing provided sufficient space for sperm movement, preventing them from escaping due to excessive motility. Based on data analysis, the optimal condition for sperm capture was as follows: sperm capture space of 70 μm, applied voltage set to 20 Vpp, and applied frequency set to 3 MHz. Under this condition, the sperm capture efficiency could reach 59.98% ± 0.93%. These results demonstrated the high application potential of the 3D TiN nano-electrode arrays in sperm capture. In the future, we hope to further optimize the parameters and process design for sperm capture, and this chip holds the potential to enhance semen quality and advance the development of assisted reproductive technologies.

## Materials and methods

### Fabrication of 3D TiN nano-electrode arrays

**FIGURE 2:**
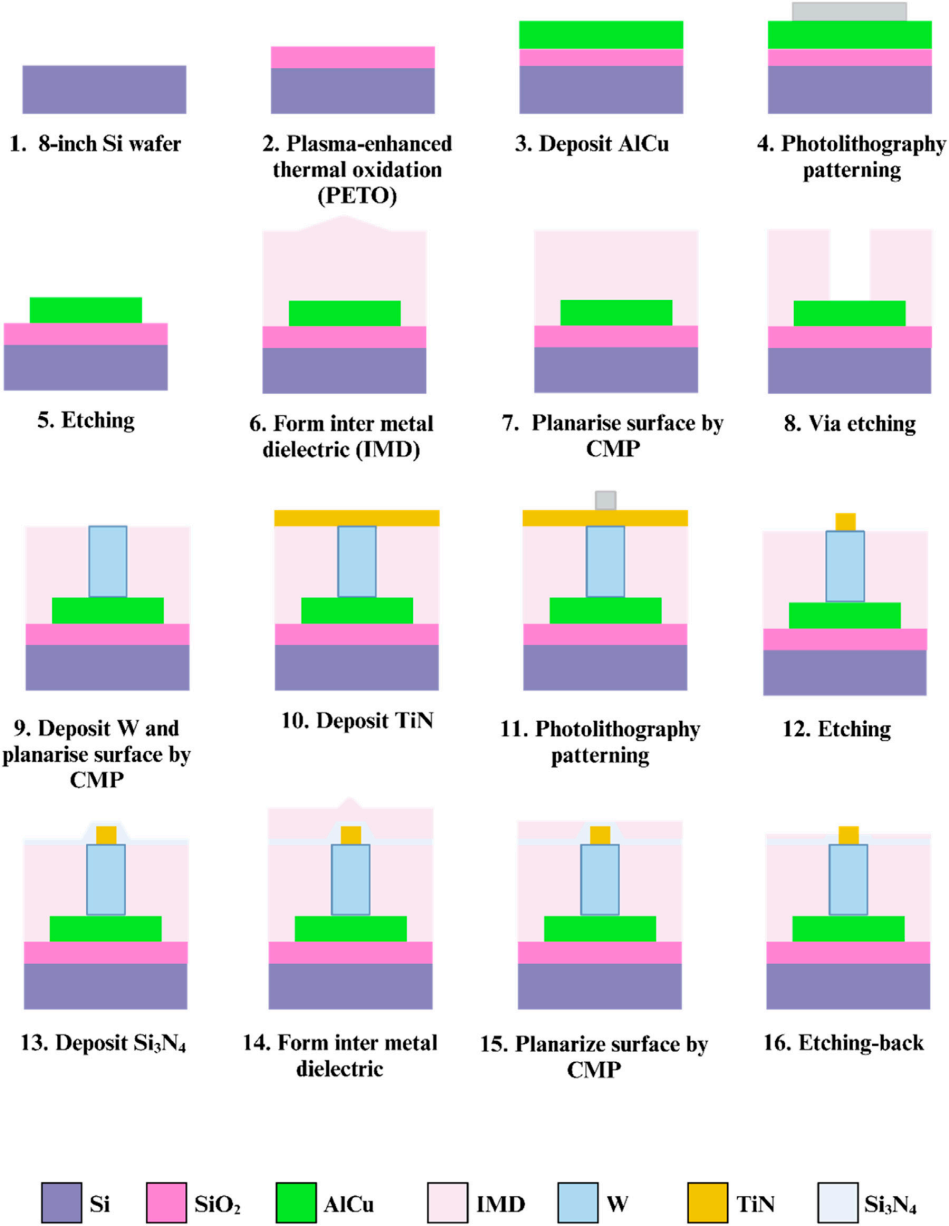
Fabrication process of the 3D TiN nano-electrode arrays.

We improved the CMOS fabrication process of the previous 3D TiN nano-electrode arrays (Lu et al., 2024), with the primary goal of reducing the potential for exposed wires due to process variations, as shown in Figure 2. In the modified fabrication process, the thin film stacking steps in the early stages of the chip remained unchanged. On an 8-inch Si wafer, we first formed a SiO_2_ layer as the substrate using thermal oxidation, then deposited AlCu using physical vapor deposition (PVD), followed by patterning and etching to create the wire structure (steps 1–5). Unlike the previous process, we added a step to fill the inter-metal dielectric (IMD) before the TiN electrodes deposition and etching. After completing the IMD filling and planarization using Chemical-Mechanical Polishing (CMP), via etching was performed, followed by deposition of tungsten with good conductivity as the connection material between the two metals (steps 6–12). Next, we deposited Si_3_N_4_ and IMD as the insulating layer, and finally, we used an etching-back technique to remove the excess insulating layers, which formed the 3D protruding cylindrical nano structure of the TiN electrodes and exposed them in the dielectric (steps 13–16). Before the experiment, we first cleaned the chips with deionized water and 95% alcohol, then dried the droplets with a nitrogen gun and set the chips aside for use.

**FIGURE 3:**
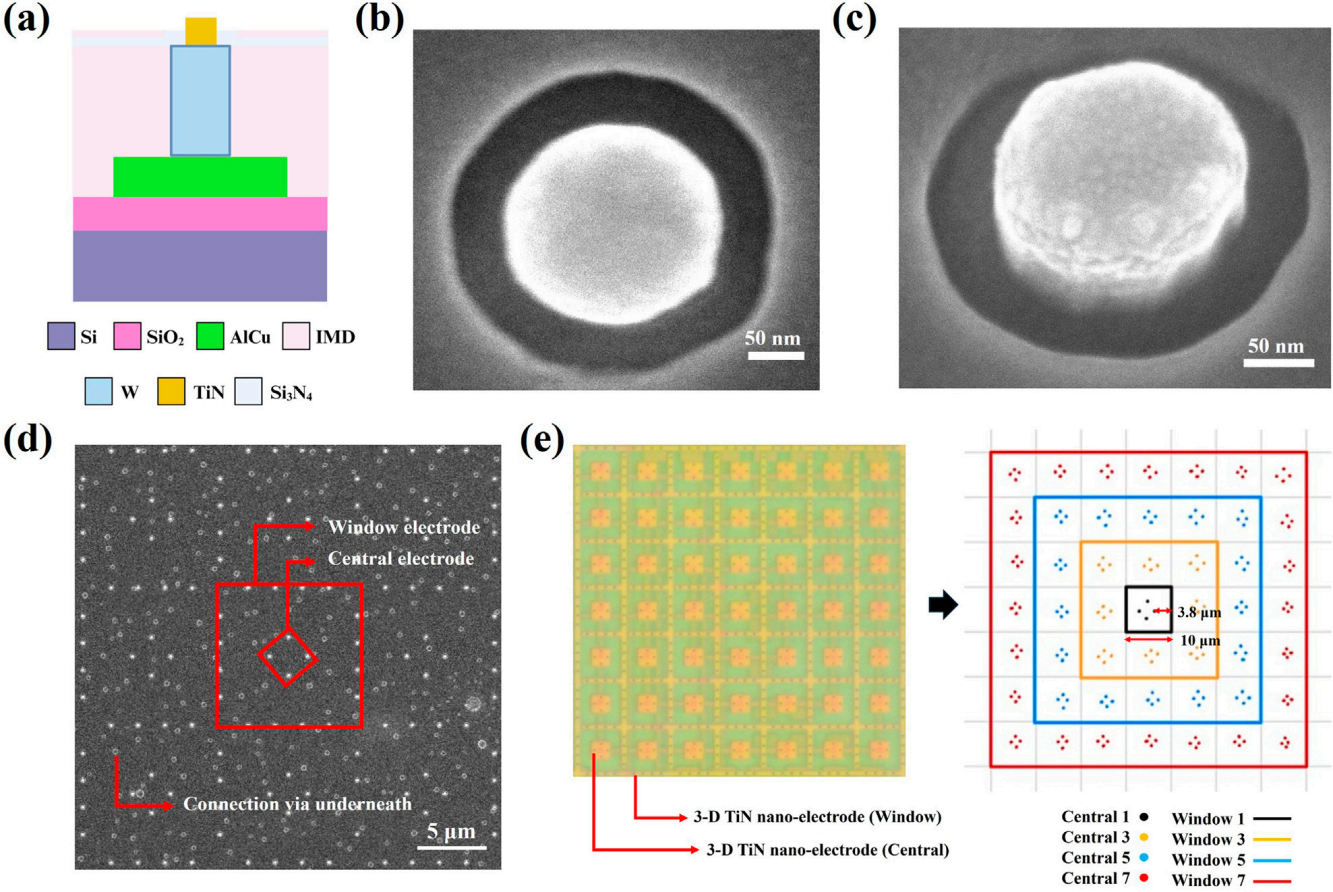
The 3D TiN nano-electrode structure of the 3D TiN nano-electrode arrays. **(a)** Cross-sectional schematic of single 3D TiN nano-electrode. **(b)** Top view of the nano-electrode captured via scanning electron microscopy. **(c)** 20° tilt view of a nano-electrode captured via scanning electron microscopy. **(d)** The arrangement of the 3D TiN nano-electrodes captured via scanning electron microscopy. **(e)** Electrode control unit of the 7 × 7 block.

Figure 3a presents a cross-sectional schematic of a single electrode, clearly illustrating that our nano-electrodes protrude from the chip surface, while the wires (AlCu) are completely buried within the chip by IMD and Si_3_N_4_ layers. The electrodes (TiN) are connected to the wires (AlCu) through tungsten. Figures 3b, c show the top view and a 20° tilted view of the chip under SEM observation, respectively. Both images reveal that the electrodes are protruding cylindrical structures. By increasing the thickness between the wiring and the chip surface while maintaining a nano-electrode diameter of 0.2 μm, we effectively avoid the issue of wire exposure caused by process variations. Additionally, we effectively reduce the generation of Joule heat by minimizing the contact area between the electrodes and medium, as well as between the wiring and medium. Rough calculations indicate that the electrode pattern density on our chip is approximately 0.37%. In contrast, the exposed area of electrodes and wiring in conventional MEMS-fabricated chips is about 840 times larger than that of our chip (Lu et al., 2024). Therefore, our design further protects sperms from thermal damage while enhancing the efficiency of dielectrophoretic capturing.

### 3D TiN nano-electrode arrays with adjustable electrode spacing and wire connections

Figure 3d illustrates the arrangement of the electrodes. These electrodes are regularly arranged in square units measuring approximately 10 μm × 10 μm. Within each square unit, the spacing between the inner electrodes and outer electrodes is about 3.8 μm, and an uneven electric field required for dielectrophoresis is generated by varying the number of inner and outer ring electrodes. Here, we define the inner ring electrodes as “Central” and the outer ring electrodes as “Window”. The actual electrode control unit consists of a 7 × 7 square block, as shown in Figure 3e, where the left side displays the appearance of the block under an optical microscope, and the right side provides an illustration explaining the control mechanism for the electrode block. This design allows for flexible selection of different combinations of inner (Central) and outer (Window) electrodes, and enables us to create dielectrophoretic regions of different extents while selecting different electrode spacing combinations. In Figure 3d, pastel circular dots represent buried connection vias located beneath the surface.

**FIGURE 4:**
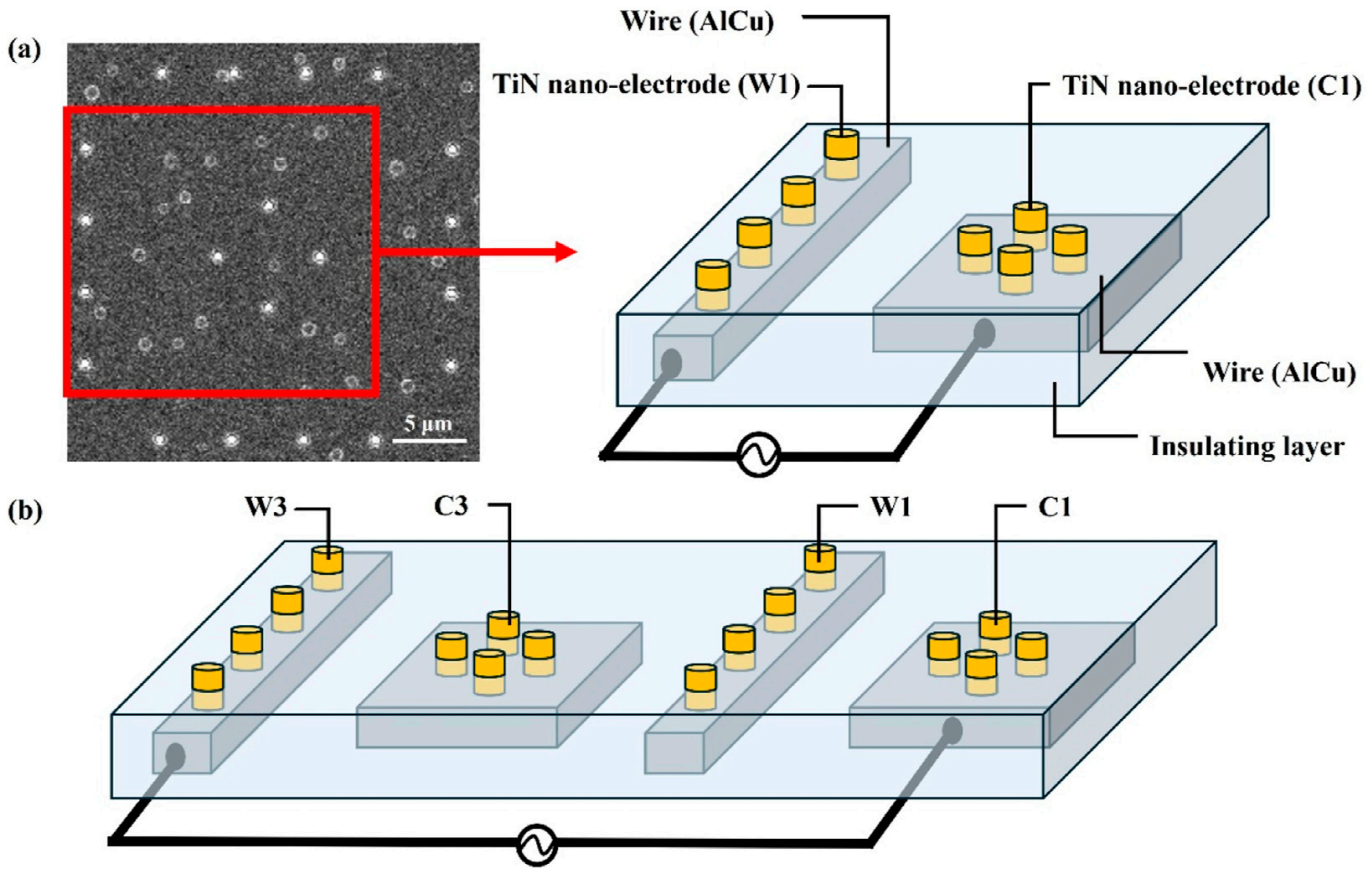
The wire connections between the inner and outer electrodes. **(a)** The wire connection between the inner electrode Central 1 (C1) and the outer electrode Window 1 (W1), creating the minimum electrode spacing of 3.8 μm. **(b)** The wire connection between Central 1 (C1) and Window 3 (W3), resulting in an electrode spacing of 13.8 μm.

Figure 4 further explains the wire connections between the inner and outer electrodes. The inner electrodes (Central) and outer electrodes (Window) are connected using different sets of wires, allowing adjustments to the electrode spacing by selecting various combinations of inner and outer electrodes. Figure 4a illustrates the wire connection between Central 1 (C1) and Window 1 (W1), creating the minimum electrode spacing of 3.8 μm. In contrast, Figure 4b shows the wire connection between Central 1 (C1) and Window 3 (W3), resulting in an electrode spacing of 13.8 μm. Additionally, an alternating current is applied between the inner and outer electrodes to generate the non-uniform electric field required for dielectrophoretic force.

### Sperm preparation

This study utilized Landrace boar semen purchased from the Animal Technology Research Institute (Miaoli, Taiwan) for experiments. Initially, sperm motility and concentration were measured using iSperm (Aidmics Biotechnology, Taipei, Taiwan) to ensure that the average motility of the stock semen exceeded 70%. To reduce the conductivity of the stock semen, it was first equilibrated in a water bath at 37°C and then gradually diluted with dielectrophoresis buffer [10 mM HEPES, 0.1 mM CaCl_2_, 236 mM sucrose, and 59 mM dextrose in deionized water, with the pH adjusted to 7.4 (Pendharkar et al., 2022)] to a final concentration of 7 × 10^6^ sperms/mL. The average motility of the diluted semen ranged between 30% and 40%, as shown in Supplementary Table S1. We speculate that the decrease in motility may be related to the dilution effect (Centurion et al., 2003). However, given that the experiment lasted only 30 s, we consider that the dilution with dielectrophoresis buffer would have a minimal impact on sperm motility during our experiments.

### Conductivity measurement

The diluted semen was measured using a Bante540 conductivity meter (Sugar land, United States) to ensure that the conductivity remained between 0.5 and 0.8 mS/cm to avoid high conductivity affecting the effectiveness of the dielectrophoretic force.

### Sperm capture via dielectrophoresis

Precisely 20 μL of diluted semen was placed onto the chip and covered with a coverslip. A function generator (MFG-2260MFA, Gwinstek, New Taipei City, Taiwan) was activated to generate a sine wave signal, generating dielectrophoretic force to capture sperms for 30 s. During this process, we adjusted the dielectrophoretic parameters, such as applied frequencies, applied voltages, and electrode spacing. A microscope (OLYMPUS MX50, Japan) was used to observe the sperm capture process and record videos to analyze difference in sperm behavior before and after dielectrophoresis. Approximately 200–300 sperms were observed per trial, and the number of the captured sperms was manually counted using MicroCamV8 software (M&T OPTICS, Taipei, Taiwan), and the sperm capture efficiency was then calculated. Sperms observed hovering in the effective area or spinning while fixed on the electrode under dielectrophoresis in the video were considered captured. The formula for calculating the sperm capture efficiency follows:

$$\text{Dielectrophoretic capture efficiency of sperms }\left(\%\right)=\frac{\left(\text{Number}\;\text{of}\;\text{captured}\;\text{sperms}\;\text{in}\;\text{dielectrophoresis}\;\text{buffer}\right)}{\left(\text{Total}\;\text{live}\;\text{sperms}\;\text{in}\;\text{dielectrophoresis}\;\text{buffer}\right)}\times 100\%$$

### Data analysis of sperm capture efficiency

The data in this study were analyzed using GraphPad Prism version 8.4.2 (GraphPad Software, CA, United States). To avoid bias in capture efficiency caused by significant individual differences between boars, all sperm capture efficiencies were normalized before comparison. The normalization baseline was set using the conditions of 3 MHz, 9 Vpp, and electrode spacing defined by open Window 7 and Central 1 (W7+C1) parameters as 100%.

One-way ANOVA was used to compare capture efficiency differences under different parameters, and the results are presented in bar charts. The vertical axis represents the capture efficiency ratio, while the horizontal axis represents different experimental groups (e.g., different applied voltages, applied frequencies, or electrode spacing). In this study, a p-value of less than 0.05 was considered statistically significant.

## Results and discussion

### Temperature increases and Joule heating of 3D TiN nano-electrode arrays during dielectrophoretic capture

We determined the current of our 3D TiN nano-electrode arrays during operation through the CV curve, showed that under a 20 Vpp voltage, the current was $7.525\times 10^{-9}$ amperes. We then obtained the Joule heat of 100 mm^2^ nano-electrode arrays, approximately $3.786\times 10^{-8}$ watts through calculation. We also used an infrared thermometer to measure the temperature increase of each 20 μL sample under dielectrophoresis for 30 s, 1 min, and 2 min (Supplementary Table S2). Compared to the micro-electrodes, our 3D TiN nano-electrode arrays exhibited a low temperature difference after 30 s of operation (0.33 °C versus 3.13°C ) (Seki and Tada, 2024). Furthermore, for the sperm capture, the operating duration was about 30 s, and a 9 Vpp voltage was applied. The temperature increase before and after the sperm capture was minimal, meaning that the impact of Joule heating on sperms can be negligible.

### Sperm morphology under the influence of dielectrophoretic force

Supplementary Movie S1 demonstrated the effect of dielectrophoretic force on the sperms in different states. From the video, it was observed that sperms in different states exhibited distinct behavioral characteristics under a dielectrophoretic force. Dead sperms, lacking motility, were not significantly affected by the dielectrophoretic force and merely floated along with the liquid flow during its application. Vigorous sperms, while influenced by the dielectrophoretic force, often managed to escape its constraints due to their strong motility. In contrast, motile sperms were more susceptible to the effects of dielectrophoretic force, becoming limited to a specific area. This phenomenon highlighted the potential of the 3D TiN nano-electrode arrays to distinguish sperm states within a semen sample. It noted that either motile sperms or vigorous sperms that we observed would be considered with good motility. Moreover, when sperms were captured by the dielectrophoretic force, they rotated within the dielectrophoretic active region. This phenomenon might be related to the shape of the sperms. Since sperms does not have a perfectly spherical shape, their head and tail exhibit significant morphological differences. This leads to varying torques and angular velocities under the influence of dielectrophoretic force, resulting in rotational motion within the dielectrophoretic region (Hu et al., 2025). After making the preliminary observation of sperm behavior under dielectrophoresis, we further evaluated the impact of different parameters on the sperm capture efficiency.

### The influence of applied voltage and applied frequency on sperm capture efficiency

**FIGURE 5:**
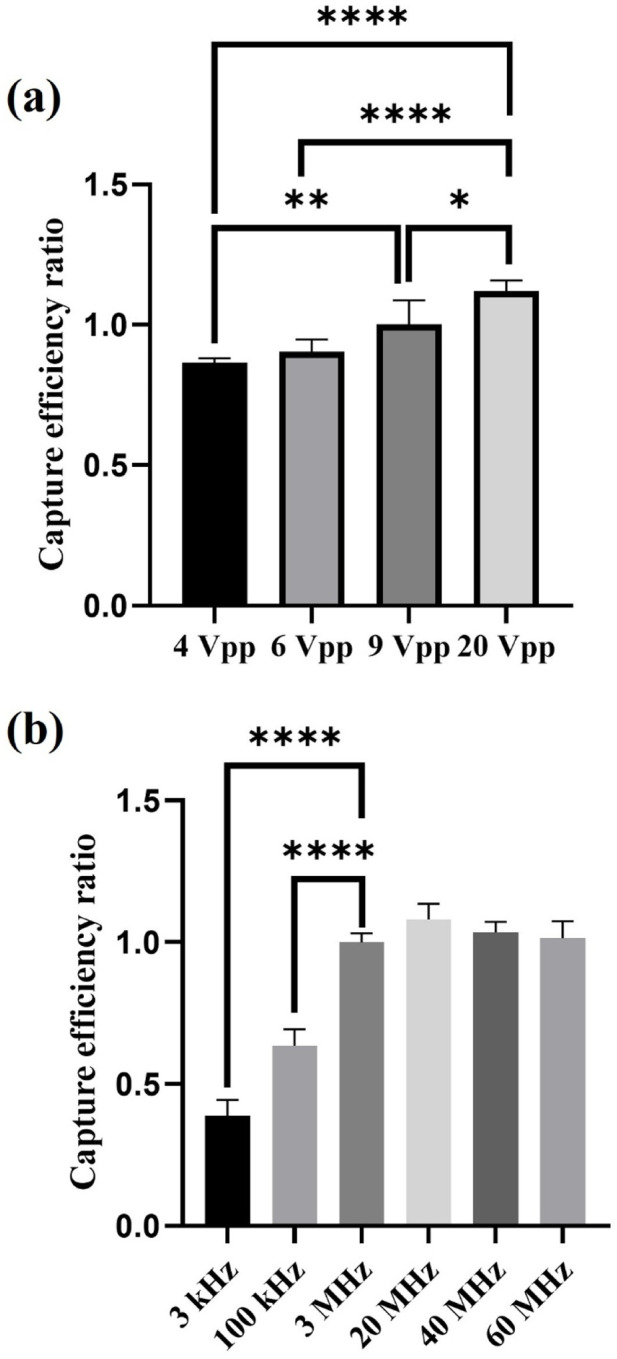
The effect of applied voltages and applied frequencies on sperm capture efficiency. **(a)** Experimental results of sperm capture efficiency at different applied voltages (n = 5). **(b)** Experimental results of sperm capture efficiency at different applied frequencies (n = 5). *, 0.01 < P < 0.05; **, 0.001 < P < 0.01; ****, P < 0.0001.

Per Formula 1, an increase in applied voltage causes the dielectrophoretic force to grow exponentially (Khouzestani et al., 2023). Theoretically then, sperm capture efficiency should increase as the applied voltage rises. In our experiment, we fixed the applied capture frequency at 3 MHz and open Window 7 and Central 1 electrodes (W7+C1), applying voltages of 4, 6, 9, and 20 Vpp for sperm capture. The results showed that the sperm capture efficiency indeed increased with the applied voltage, which aligned with our expectation. However, the growth in sperm capture efficiency did not exhibit an exponential increase pattern matching the rise in dielectrophoretic force, as shown in Figure 5a. We believe the reason for the lack of exponential growth in sperm capture efficiency might be related to sperm motility and individual differences. Although motile sperms were captured under the influence of dielectrophoretic force, compared to bacteria or cells, sperms possess stronger energy and motility. Therefore, sperms are more capable of resisting the dielectrophoretic force and escaping. In this study, the capture efficiency reached 59.98% ± 0.93% at 20 Vpp, which was the highest sperm capture efficiency observed so far. However, when testing the effect of different voltages in dielectrophoretic capture on sperm viability, we found that after being captured at 20 Vpp, sperm viability decreased significantly compared to 9 Vpp. This indicated that the chip still caused some damage to sperm under high-voltage capture. In contrast, using 9 Vpp achieved effective capture while minimizing damage to sperms. Therefore, in subsequent experiments, we selected 9 Vpp as the control voltage for testing other parameters. The relevant data on sperm viability is provided in Supplementary Table S4.

Figure 5b shows the results of the effect of different applied frequencies on sperm capture efficiency. In this experiment, we fixed the applied capture voltage at 9 Vpp and opened Window 7 and Central 1 electrodes (W7+C1) to test sperm capture efficiency at applied frequencies of 3 kHz, 100 kHz, 3 MHz, 20 MHz, 40 MHz, and 60 MHz. Per Formula 1, all factors remained the same other than applied frequency, so only the frequency-dependent CM factor would change, thereby affecting the magnitude of the dielectrophoretic force. Based on the relationship between frequency and the real part of the CM factor, we can make the following predictions (Park et al., 2020): 1) At low frequencies, the real part of the CM factor is smaller, resulting in weaker dielectrophoretic force; conversely, at higher frequencies, the dielectrophoretic force is stronger. Therefore, theoretically, the sperm capture efficiency at high applied frequencies should be higher than at low applied frequencies. 2) When the applied frequency reaches a certain optimal high applied frequency, the real part of the CM factor reaches its maximum, the curve enters a plateau state, and the dielectrophoretic force remains at its maximum value. The sperm capture efficiency also reaches its highest value and the growth trend flattens out. As the applied frequency increases further, the real part of the CM factor starts to decline, the dielectrophoretic force weakens, and the sperm capture efficiency decreases. These predictions align with our experimental results.

In our experimental parameters, applied frequencies of 3, 20, 40, and 60 MHz fall within the range of 10^6^–10^7^ Hz, where the real part of the CM factor is in the plateau state of the curve (Park et al., 2020). Applied frequencies of 3 kHz and 100 kHz correspond to 10^3^ Hz and 10^5^ Hz, respectively, where the real part of the CM factor curve shows an increasing trend. The experimental results indicated that sperm capture efficiency was more ideal at high applied frequencies (in the MHz range), whereas at low applied frequencies (in the kHz range), sperm capture efficiency was lower. Additionally, we observed that within the high applied frequency range, the sperm capture efficiency did not significantly change, suggesting that as the applied frequency increased further, the dielectrophoretic force reached its maximum capturing effect, and it had not yet surpassed the critical frequency at which dielectrophoretic force weakens. Therefore, the sperm capture efficiency remained consistent without a significant decrease.

### The effect of electrode spacing and capture space on sperm capture efficiency

**FIGURE 6:**
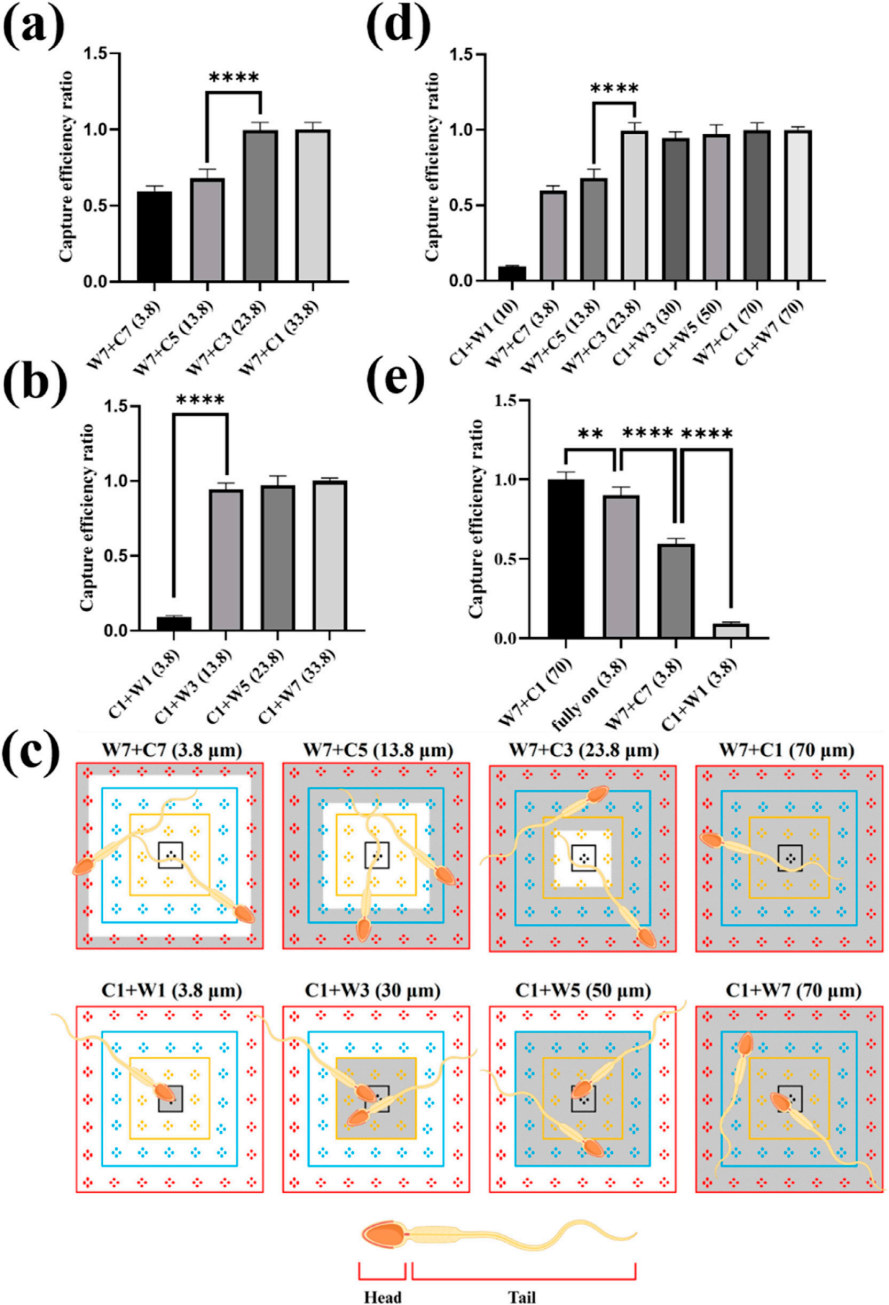
The effect of electrode spacing and sperm capture space on sperm capture. **(a)** The effect of using different inner ring electrodes (Central) paired with the outer ring electrode Window 7 (W7) on sperm capture efficiency. **(b)** The impact of using different outer ring electrodes (Window) paired with the inner ring electrode Central 1 (C1) on sperm capture efficiency. **(c)** The capture space available to sperms with different electrode combinations. The gray-shaded area represents the capture space when sperms were captured. **(d)** The effect of sperm capture space on capture efficiency. **(e)** The effect of different 3.8 μm electrode spacing combinations on sperm capture efficiency. **, 0.001 < P < 0.01; ****, P < 0.0001.

Figures 6a, b demonstrate the impact of selecting different Central and Window electrodes on sperm capture efficiency. The experimental conditions for both cases were fixed with an applied voltage of 9 Vpp and an applied frequency of 3 MHz. Figure 6a shows the effect of using different inner ring electrodes (Central) paired with the outer ring electrodes Window 7 (W7) on sperm capture efficiency. The results indicated a significant leap in sperm capture efficiency between electrode spacing of 13.8 μm and 23.8 μm. Figure 6b demonstrates the impact of using different outer ring electrodes (Window) paired with the inner ring electrodes Central 1 (C1) on sperm capture efficiency. Notably, at an electrode spacing of 13.8 μm, the sperm capture efficiency significantly increased. This differed from the sperm capture efficiency surge observed with the electrode configuration in Figure 6a, which occurred at an electrode spacing of 23.8 μm.

We further reviewed the videos of the sperm capture process (Supplementary Movies S2, S3) and found that different electrode spacing combinations created distinct “net” for sperm capture. These “nets” provided sperms with varying capture spaces, which, in turn, affect capture efficiency. This capture space corresponds to the effective area of dielectrophoresis. In an electrode control unit, a 7 × 7 block, the differing number of electrodes between the inner and outer ring electrodes created a non-uniform electric field, causing sperms passing through this region to be affected by dielectrophoretic force. Therefore, when different electrode combinations were connected, each 7 × 7 block generated a different capture pattern, whose pattern could be predicted based on the position of the inner and outer ring electrodes. For example, when we chose the C1+W7 electrode combination, it created a dielectrophoretic region that occupied the entire block. Sperms passing through this area experienced dielectrophoretic force and were confined within this “net.” Figure 6c illustrates the sperm capture space provided by different electrode combinations, where the gray-shaded area represents the space where sperms are captured. The dynamic behavior is shown in Supplementary Movie S4. Based on these observations, we defined the sperm capture space as a variable, as shown in Figure 6d. From the figure, we observed that when the sperm capture space reached 23.8 μm, the sperm capture efficiency increased significantly. Further analysis revealed that as the capture space increased, there was no significant improvement in sperm capture efficiency. These results suggested that the sperm capture space must not exceed a specific range to achieve optimal capture efficiency. Beyond this range, increasing the capture space further did not significantly enhance capture efficiency.

It was clear that selecting an appropriate “net” for sperm capture was crucial for improving the capture efficiency. We believed that the high motility of sperms prevented them from being completely immobilized by dielectrophoresis like bacteria or cells. However, as observed in our movies, the sperms could indeed be limited within a certain region by the dielectrophoretic force. This “net” for capturing sperms should not be too small, and its size was clearly related to the sperm size and the capture space. Although previous studies had confirmed that dielectrophoresis affected the head and tail of the sperm differently (Shuchat et al., 2019), our results indicated that the head was more affected by the dielectrophoretic force. According to Formula 1, the larger the volume, the greater the dielectrophoretic force experienced. Since the volume of the sperm head was larger than that of the flagellum, the sperm head was subject to a relatively greater dielectrophoretic force. Furthermore, literature indicated that the magnitude of the dielectrophoretic force was also influenced by the shape of the particle; the more a particle deviated from a spherical shape, the weaker the dielectrophoretic force it experiences (Li et al., 2018). Based on this, it could be inferred that the elliptical-shaped sperm head experienced a stronger dielectrophoretic force compared to the elongated sperm tail. Our results showed that when the “net” for capturing sperms had a capture space of 23.8 μm, the capture efficiency significantly improved. Considering the size of the sperm, the head of the Landrace sperms was approximately 8–10 μm long (Szablicka et al., 2022), and a 23.8 μm range roughly covered the sperm head and a part of the flagellum, covering about half of the total sperm length. This range provided sufficient space for the head movement, enabling effective sperm capture under these conditions.

On the other hand, we compared three electrode spacing combinations, all with a 3.8 μm spacing, which showed significant differences in sperm capture efficiency, as shown in Figure 6e. We attributed these differences to the varying sizes of the dielectrophoretic effective areas. As illustrated in Supplementary Figure S2, when the electrodes were fully open, the dielectrophoretic effective area covered the entire working region. The effective area where the electrodes were fully open was about 2 times larger than the effective area open Window 7 and Central 7 (W7+C7) and 49 times larger than the effective area open Central 1 and the Window 1 (C1+W1), with the effective area was roughly estimated based on the number of square units in the active region, for example, the effective area of the fully open electrodes was 49 square units. The smaller the effective area, the lower the probability of sperms passing through the dielectrophoretic zone, which led to decreased sperm capture efficiency. Therefore, the size of the dielectrophoretic effective area affected the likelihood of sperm captured. However, from the comparison between electrodes fully open and open Window 7 and Central 1 (W7+C1) combination, we found that electrode spacing had a greater impact on sperm capture efficiency than the effective area of the electrodes. We discovered that, although both combinations covered the entire working area, the sperm capture efficiency with the open Window 7 and Central 1 (W7+C1) combination was significantly higher than that of the fully open electrodes. This further highlighted the importance of selecting the “appropriate net” for capturing sperms. Through comparisons between Supplementary Movies S2, S5 showing sperm capture, we observed that sperms were influenced by multiple dielectrophoretic electric fields simultaneously, potentially causing them to jump between different effective areas of dielectrophoresis. This demonstrated the contrast between “net” capture and fully open electrodes capture techniques.

By adjusting the electrode spacing within 3D TiN nano-electrode arrays, we were able to accommodate the sperm sizes of different species, thereby optimizing sperm capture efficiency and enhancing the versatility of our 3D TiN nano-electrode arrays chip for various applications. Our findings suggested that setting the capture range to at least half the sperm length effectively confined motile sperms for efficient capture. For instance, horse sperm, typically 61–86 μm in length (Varner et al., 2015), was best suited for electrode spacing combinations such as C1+W5 (capture space 50 μm) or C1+W7 (capture space 70 μm). However, if the sperm length exceeded the adjustable range of the chip, for example, the sperm of a honey possum was approximately 349 μm (Thompson et al., 2018), and our dielectrophoresis chip with an effective capture was more challenging.

### Throughput of handling sperms with 3D TiN nano-electrode arrays

Despite the numerous promising dielectrophoretic sperm separation devices available, throughput is often a significant practical application challenge. To address this issue, the chip used in this study employs a large-scale processing region instead of a conventional channel structure processing region. This not only simplifies the device design but also significantly increases sample throughput. Currently, a single chip can process up to 50 μL of sample at a time, with the dielectrophoretic capture time set to 30 s. As a result, the maximum throughput of a single chip can reach 6 mL/h, which is 6–100 times higher than other dielectrophoretic sperm capture devices (Dararatana et al., 2015; Wongtawan et al., 2020). Additionally, this chip offers good scalability, allowing for parallel operation with multiple chips to further increase throughput based on sample demand.

## Conclusion

In this study, we mainly explored the impact of various dielectrophoretic parameters on motile sperm capture efficiency. Our 3D TiN nano-electrode arrays fabricated through using CMOS technology, which not only reduced the negative impact of Joule heating on sperm capture but also featured a strong electric field, scalability, and adjustable electrode spacing, allowing the applications of our dielectrophoresis chip to be more flexible.

In the experiment, we investigated the effects of applied voltages, applied frequencies, and adjustable electrode spacing on sperm capture efficiency. The results showed that sperms were captured by dielectrophoretic force spin within the dielectrophoretic region, a phenomenon related to the sperm morphological characteristics, which affected their torque and angular velocity. Furthermore, the sperm motility was found to be a crucial factor influencing whether the sperms could be captured. Although increasing applied voltage did improve sperm capture efficiency, we found that the increase was not proportional to the exponential growth of dielectrophoretic force. This was related to variations in sperm motility. Regarding applied frequency, we discovered that selecting an appropriate applied frequency (MHz) maximized the dielectrophoretic force, thus enhancing sperm capture efficiency. Interestingly, we also observed that an appropriately sized “net” could effectively restrict the sperms within the dielectrophoretic region, and the size of the “net” needed to match the collective sperm size. A larger net did not significantly improve sperm capture efficiency. Using the techniques described, we have achieved a sperm capture efficiency of 59.98% ± 0.93%, under conditions of sperm capture space of 70 μm, applied voltage of 20 Vpp, and applied frequency of 3 MHz. Under these conditions, the throughput of single-chip processing could reach 6 mL/h, and it could be further increased via parallel operation while using multiple chips.

## Data Availability

The original contributions presented in the study are included in the article/Supplementary Material, further inquiries can be directed to the corresponding author.
