# Supplementary material for: Innovative CMOS-fabricated dielectrophoretic chip: application of 3D TiN nano-electrode arrays with adjustable electrode spacing in sperm capture
Source: Front Bioeng Biotechnol. 2025 Mar 27;13:1565743. doi: 10.3389/fbioe.2025.1565743 (PMC11983159; doi:10.3389/fbioe.2025.1565743)
Supplement: Supplementary file 6 [file DataSheet1.docx]

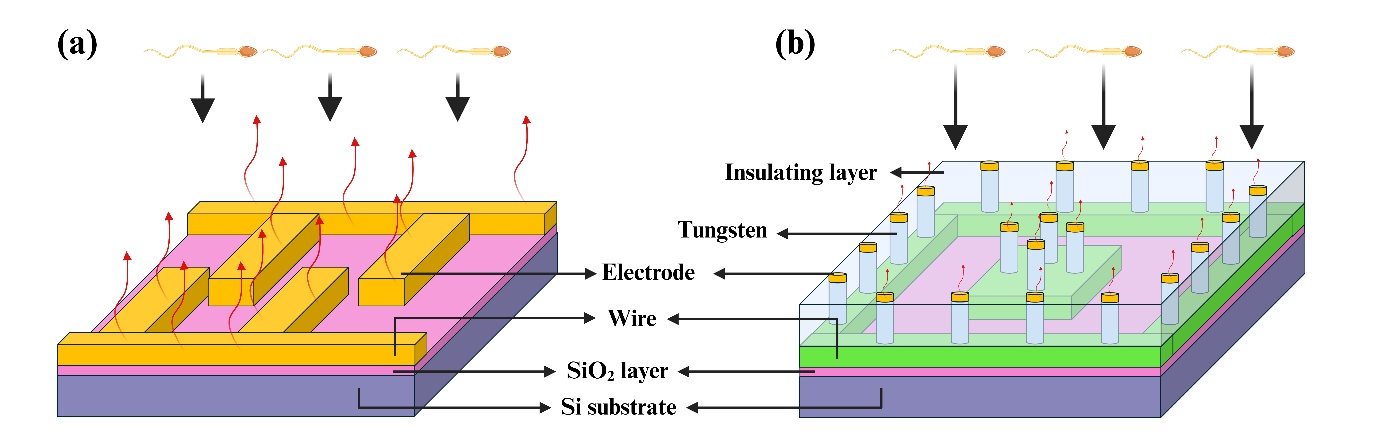


**Figure S1.** Schematic comparison of the generation of Joule heating via (a) micro-electrode and (b) nano-electrode device.


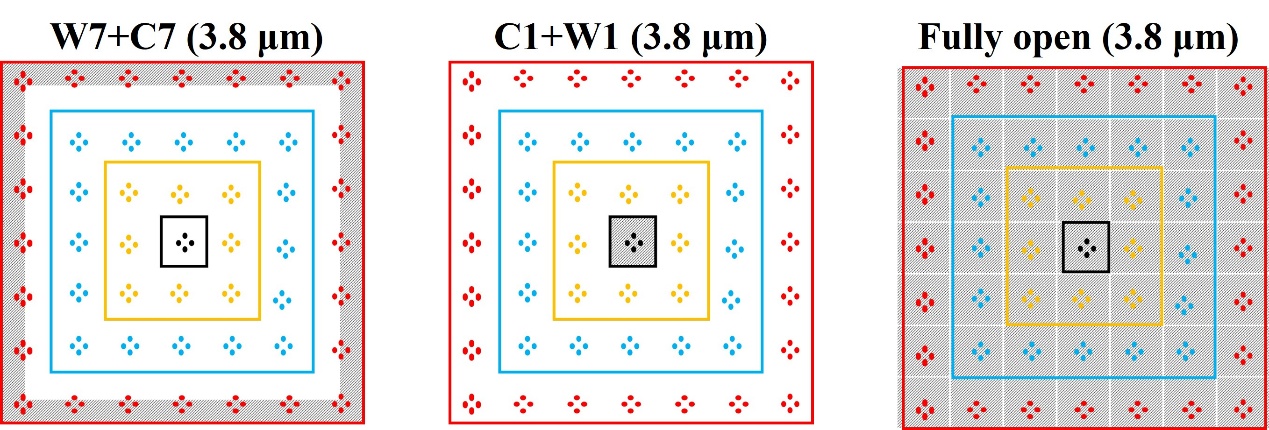


**Figure S2.** Schematic of the dielectrophoretic effective areas for different combinations of 3.8 μm electrode spacing.

**Table S1.** Relationship between the sperm concentration and motility.

|  | Concentration (n=3) | Motility (n=3) |
| --- | --- | --- |
| Stock semen | $1.1\times{10}^{8}sperm/mL$ | More than 70% |
| 2-fold dilution (1/2) | $0.61\times{10}^{8}sperm/mL$ | More than 70% |
| 8-fold dilution (1/8) | $0.12\times{10}^{8}sperm/mL$ | 30%~40% |

**Table S2.** The experimental data of the temperature increase measured via an infrared thermometer while the dielectrophoretic capture at 9 or 20 Vpp for 30 seconds, 1 minute and 2 minutes.

|  | 9 Vpp (n=3) | 20 Vpp (n=3) |
| --- | --- | --- |
| 30 seconds | 0.33 $℃$ | 0.86 $℃$ |
| 1 minute | 0.43 $℃$ | 1.53 $℃$ |
| 2 minutes | 0.63 $℃$ | 1.96 $℃$ |

**Table S3.** Sperm viability while the dielectrophoretic capture at different applied voltages.

|  | 0 Vpp | 3 MHz, 9 Vpp | 3 MHz, 20 Vpp |
| --- | --- | --- | --- |
| Viability | More than 90% | More than 90% | 80%~90% |
